# A clinical and EEG scoring system that predicts early cortical response (N20) to somatosensory evoked potentials and outcome after cardiac arrest

**DOI:** 10.1186/1471-2261-8-35

**Published:** 2008-12-04

**Authors:** Cédric Daubin, Damien Guillotin, Olivier Etard, Cathy Gaillard, Damien du Cheyron, Michel Ramakers, Bruno Bouchet, Jean-Jacques Parienti, Pierre Charbonneau

**Affiliations:** 1 Department of Medical Intensive Care, Avenue Côte de Nacre, Caen University Hospital, 14033 Caen Cedex, France; 2 Laboratory of neurological functional exploratory, Avenue Côte de Nacre, Caen University Hospital, 14033 Caen Cedex, France; 3 Department of Biostatistics and Clinical Research, Avenue Côte de Nacre, Caen University Hospital, 14033 Caen Cedex, France; 4 Inserm UMR-S 707, Paris, F-75012, Université Pierre et Marie Curie-Paris6, UMR-S 707, Paris, F-75012, France

## Abstract

**Background:**

Anoxic coma following cardiac arrest is a common problem with ethical, social, and legal consequences. Except for unfavorable somatosensory-evoked potentials (SSEP) results, predictors of unfavorable outcome with a 100% specificity and a high sensitivity are lacking. The aim of the current research was to construct a clinical and EEG scoring system that predicts early cortical response (N20) to somatosensory evoked potentials and 6-months outcome in comatose patients after cardiac arrest.

**Methods:**

We retrospectively reviewed the records of all consecutive patients who suffered cardiac arrest outside our hospital and were subsequently admitted to our facility from November 2002 to July 2006. We scored each case based on early clinical and EEG factors associated with unfavorable SSEPs, and we assessed the ability of this score to predict SSEP results and outcome.

**Results:**

Sixty-six patients qualified for inclusion in the cohort. Among them, 34 (52%) had unfavorable SSEP results. At day three, factors independently associated with unfavorable SSEPs were: absence of corneal (14 points) and pupillary (21 points) reflexes, myoclonus (25 points), extensor or absent motor response to painful stimulation (28 points), and malignant EEG (11 points). A score >40 points had a sensitivity of 85%, a specificity of 84%, and a positive predictive value (PPV) of 85% to predict unfavorable SSEP results. A score >88 points had a PPV of 100%, but a sensitivity of 18%. Overall, this score had an area under ROC curves of 0.919. In addition, at day three, a score > 69 points had a PPV of 100% with a sensitivity of 32% to predict death or vegetative state.

**Conclusion:**

A scoring system based on a combination of clinical and EEG findings can predict the absence of early cortical response to SSEPs. In settings without access to SSEPs, this score may help decision-making in a subset of comatose survivors after a cardiac arrest.

## Background

Despite improvements in cardiopulmonary resuscitation, 56% to 90% of patients who remain comatose after cardiac arrest have a poor outcome (death or permanent vegetative state) [1]. For this reason, intensive care physicians are confronted with the ethical question of whether to continue treatment. In this context, early predictors of poor outcome would be valuable.

In the last few decades, several clinical and electrophysiological variables have been reported to be strongly associated with a poor outcome in comatose survivors of cardiac arrest; these include absence of pupillary and corneal reflexes, absent motor response to pain [2-5], myoclonus or epilepticus status [6,7], an increase of neuron specific enolase (NSE) in serum [8], and a burst-suppression or isoelectric electro-encephalography (EEG) pattern [7,9,10]. However, in most cases, evidence predictive of poor outcome remains to be determined [1,8,11].

In contrast, bilateral absence of early cortical responses to somatosensory evoked potentials (SSEPs) demonstrated a 100% specificity for predicting poor outcome in this population [1,5,8,12-17], but this electrophysiological procedure is not routinely performed in all ICUs. For example, in a systematic review of early prediction of poor outcome in anoxic-ischaemic coma, the prognostic value of SSEPs was studied in only 11 out of 33 studies (33%) [1]. Therefore, alternatives are needed in settings without SSEPs.

We identified risk factors associated with unfavorable SSEP results; and based on these risk factors, we computed an early clinical and EEG score and assessed its predictive value, sensitivity, and specificity for predicting unfavorable SSEP results. Finally, we assessed the value of this score for predicting poor neurological outcomes in this patient population.

## Methods

### Patients

We conducted a retrospective cohort study of all consecutive patients who had suffered cardiac arrest outside the hospital and were subsequently admitted to the adult intensive care unit in the Caen University Hospital from November 2002 to July 2006. Patients who died or awoke within the first three days of admission were excluded from this analysis.

### Data collection

The institutional review board was consulted and considered that this protocol did not require Ethical approval given the observational and non-interventional nature of the study investigating routine care. Clinical variables collected at baseline were: age, sex, medical history (cardiovascular, respiratory, neurologic, metabolic diseases), cause of the arrest (cardiac, respiratory, other or unknown), time between arrest and cardiopulmonary resuscitation (<3 min, 3–5 min, >5 min), cardiac rhythm before cardiopulmonary resuscitation (ventricular fibrillation or tachycardia, asystole, pulseless rhythm), duration of cardiopulmonary resuscitation (<5 min, 5–15 min, >15 min), and scoring of disease severity within the first day in ICU as assessed by admission Simplified Acute Physiology Score type II (SAPS II) [18] and Acute Physiology and Chronic Health Evaluation (APACHE) II score [19].

Clinical procedures performed at days one and three were: pupillary light reflex (present/absent), motor response to painful stimulation (extensor or absent response/other response), corneal reflex (present/absent), tonic-clonic seizures (present/absent) and myoclonus (present/absent).

### Electrophysiological assessment

EEG and SSEPs were routinely performed in our center. For the purpose of this study, a neurophysiologist expert (O.E.) retrospectively reviewed all EEGs to allow similar definitions and increase homogeneity of the material. He was blinded to the clinical features at the time of recordings and to the outcome. EEG was recorded on at least a 10-channels system with needle electrodes using 10–20 international system (Fp1, Fp2, C3, C4, T5, T6, O1, and O2). The EEG patterns were classified at day one and day three according to the classification system of Synek et al [20,21] [see Additional file 1]. EEG results were dichotomized as malignant and non-malignant, including benign and uncertain patterns. SSEPs were systematically performed the third day after cardiac resuscitation. However, if SSEPs recording was due on a weekend day, the recording was postponed to Monday. SSEps were recorded on Nicolet Viking IV using 6 channels: erb'point; C6sp; C'3 or C'4 controlateral to the stimulated hand and Fpz (ipsilateral ear was used as reference). The two remaining channels served as channels control: C'3 – C'4 (or C'3–C'4) on which N20 amplitude was measured and Fpz-C'3 (or Fpz-C'4) in order to check for long-latency component using larger time-window. Absence of early cortical responses to somatosensory evoked potentials were asserted only if the 3 following conditions were present: (i) correct peripheral (N10) and medullar (N13) component, (ii) no deflexion higher than 0.5 μV on C3–C'4 (or C'3–C'4) (iii) no late component on Fpz-C'3 (or Fpz-C'4). Two groups of patients were defined for statistical analysis according to SSEP results: Group 1, patients with bilateral absence of early cortical responses (unfavorable result of SSEPs) and Group 2, patients with uni- or bilateral presence of early cortical responses (favorable result of SSEPs).

### Assessment of outcome

Neurological status at six months after cardiac arrest was recorded using the five grade Glasgow-Pittsburgh Cerebral Performance Category (GP-CPC) scale [22] [see Additional file 2]. Neurological outcome was classified as favorable (GP-CPC 1, 2, and 3) and unfavorable (GP-CPC 4 and 5).

### Treatment and treatment restriction

All patients were treated without restriction for the first three days following cardiac arrest. They received standard intensive care management and monitoring, including induced hypothermia as recommended [23,24]. Propofol, midazolam and sufentanyl were used as standard sedative and were stopped shortly before clinical assessment and EEG recording when this was possible. Hypothermia was induced using a endovascular cooling catheter (Icy™, Alsius, Irvine, CA, USA), inserted in the inferior vena cava via the femoral vein and connected to a cooling device (Coolgard 3000™, Alsius, Irvine, CA, USA), and was maintained during 24 hours. For patients which underwent cooling after resuscitation, clinical variables and neurophysiological tests were performed after rewarning. In our practice, bilateral lack of cortical response to SSEPs leads to active care withdrawal.

### Statistical analysis

Quantitative variables were expressed as means ± standard deviation (SD) with their 95% confidence intervals (CI). Qualitative variables were expressed as percentages with their 95% CIs.

All analyses were replicated for day one and day three variables. Firstly, we investigated the univariate associations between clinical and EEG results (predictors) and outcomes (pejorative results of SSEPs and 6-month poor neurological outcome) using Fisher exact tests. Secondly, we constructed a multivariable model predicting the probability of outcome by performing a backward logistic regression that included variables associated at *p *< 0.25 in univariate analysis. Thirdly, we computed a score, based on the point system developed by Sullivan [25], using the *beta *coefficients of all risk factors that were significantly associated with outcomes in the multivariable analysis. The *beta *coefficients were multiplied by 10 to round them to the nearest integer and then were summed. Fourthly, we plotted Receiver Operating Characteristics (ROC) curves to estimate the capacity of the score to predict outcome using the Area Under Curve (c-index), sensitivity (Se) and specificity (Sp). We used SPSS 14.0 (Chicago, IL) for data analysis. All tests were two-sided and a *p*-value < 0.05 was considered statistically significant.

## Results

### Baseline characteristics and neurological outcome

**Figure 1:**
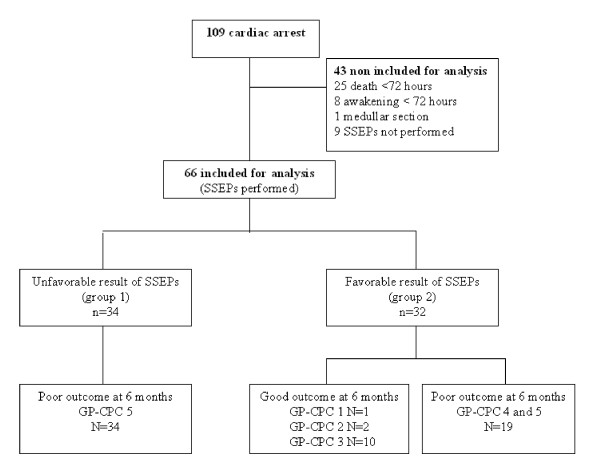
Profile of the study.

Of the 109 consecutive patients resuscitated after a cardiac arrest outside the hospital and admitted to our intensive care unit during the study period, 66 fulfilled inclusion criteria for analysis: 34 (52%) in group 1 (unfavorable result of SSEPs) and 32 (48%) in group 2 (favorable result of SSEPs), as shown in Figure 1. Patient baseline characteristics are presented in Table 1. Fifteen patients (22%) underwent cooling after resuscitation (6 in group 1 and 9 in group 2). Only asystole as cardiac rhythm before CPR (odds ratio [OR] = 0.3; 95% CI = 0.1–1; *p *= 0.04), and duration of CPR > 15 min (OR = 0.3; 95% CI = 0.1–0.9; *p *= 0.03) were significantly associated with group 1. The neurological outcome at six months was favorable in 13 (20%) patients, all in group 2 (Figure 1).

**Table 1:** Baseline characteristics of patients (n = 66).

**Characteristics**	
Age (years)	57,2 +/- 12,8
Sex ratio:% male	77%
SAPS II	63 +/- 15
APACHE II	26 +/- 7
***Medical History***	
Cardiovascular diseases n (%)	28 (42)
Neurologic diseases n (%)	5 (8)
Respiratory diseases n (%)	4 (6)
Metabolic diseases n (%)	11 (17)
***Primary cause of arrest***	
Cardiac n (%)	39 (59)
Pulmonary n (%)	16 (24)
Other or unknown n (%)^#^	11 (17)
***Time between arrest and initiation of CPR initiation of CPC***	
< 3 minutes n (%)	15 (23)
> 3 and < 5 minutes n (%)	5 (8)
> 5 minutes n (%)	34 (52)
Unknown n (%)	12 (18)
***Primary rhythm***	
Ventricular fibrillation n (%)	19 (29)
Ventricular tachycardia n (%)	3 (5)
Asystole n (%)	28 (42)
Unknown n (%)	16 (24)
***Duration of CPR***	
< 5 minutes n (%)	8 (12)
> 5 and < 15 minutes n (%)	22 (33)
> 15 minutes n (%)	31 (47)
Unknown n (%)	5 (8)

Data are presented as the mean +/- standard deviation (SD) or number (%) when appropriate.

SAPS II: Simplified Acute Physiology Score type II. APACHE II: Acute Physiology And Chronic Health Evaluation II. CPR: Cardipulmonary Resuscitation

^#^5 unknown, 3 hangings, 1 anaphylactic shock, 1 hypothermia, 1 hyperkaliemia

### Predictors of unfavorable result of SSEPs

In univariate analysis (Table 2), absence of corneal and pupillary reflexes, myoclonus, and extensor or absent motor response to painful stimulation was significantly associated with group 1 at day one and day three. A malignant EEG was also significantly associated with group 1 at day three. In multivariate logistic-regression analysis (Table 2), absence of corneal reflex, myoclonus, extensor or absent motor response to painful stimulation were independently associated with a unfavorable result of SSEPs, at both day one and day three. In addition, absence of pupillary reflex and a malignant EEG were also independently associated with group 1 at day three.

**Table 2:** Results of univariate and multivariate analysis to define predictors of bilateral absence of early cortical responses to SSEPs (group 1) at day 1 and day 3.

**n = 66**	**Univariate analysis**			**Multivariate analysis**			
	
	OR	95% CI*	p value	Beta**	OR	95% CI*	p-value
***Day-1***							
Bilateral absence of corneal reflex	6.2	[2.0–19.0]	0.001	1.0	2.7	[0.7–11.0]	0.159
Bilateral absence of pupillary light reflex	9.3	[1.9–45.5]	0.006	-	-	-	-
Presence of myoclonus	22.5	[6.0–83.8]	<0.001	3.0	20.2	[4.8–84.7]	<0.001
Tonic-clonic seizure	1.2	[0.3–4.9]	0.794	-	-	-	-
Presence of malignant EEG	3.4	[0.8–14.4]	0.090	-	-	-	-
Extensor or absent motor responses to pain	5.4	[1.3–21.8]	0.017	1.7	5.6	[0.9–32.8]	0.055
							
***Day-3***							
Bilateral absence of corneal reflex	9.9	[3.0–32.4]	<0.001	1.4	4.1	[0.8–20.9]	0.088
Bilateral absence of pupillary light reflex	21.7	[2.6–178.1]	0.004	2.1	7.8	[0.7–89.0]	0.097
Presence of myoclonus	7.9	[2.3–27.4]	0.001	2.5	11.7	[2.0–68.3]	0.006
Tonic-clonic seizure	0.2	[0.0–1.5]	0.108	-	-	-	-
Presence of malignant EEG	6.6	[1.9–23.2]	0.003	1.1	3.1	[0.6–16.2]	0.183
Extensor or absent motor responses to pain	22.6	[2.7–186.4]	0.004	2.8	17.3	[1.1–210.0]	0.025

* CI: Confidence Interval; ** Beta denotes the estimated coefficient in the multivariate logistic regression model which predicted poor SSEPs results. The score related to each item is calculated as follows: points = beta × 10, according to Sullivan et al. [25]

### Score predictive of unfavorable result of SSEPs

At days one and three, the prediction models included three and five variables, respectively: myoclonus (30 points), extensor or absent motor responses to painful stimulation (17 points), and absence of corneal reflex (10 points) for day one, and extensor or absent motor responses to pain (28 points), myoclonus (25 points), absence of pupillary (21 points) and corneal (14 points) reflexes, and a malignant EEG (11 points) for day three.

**Figure 2:**
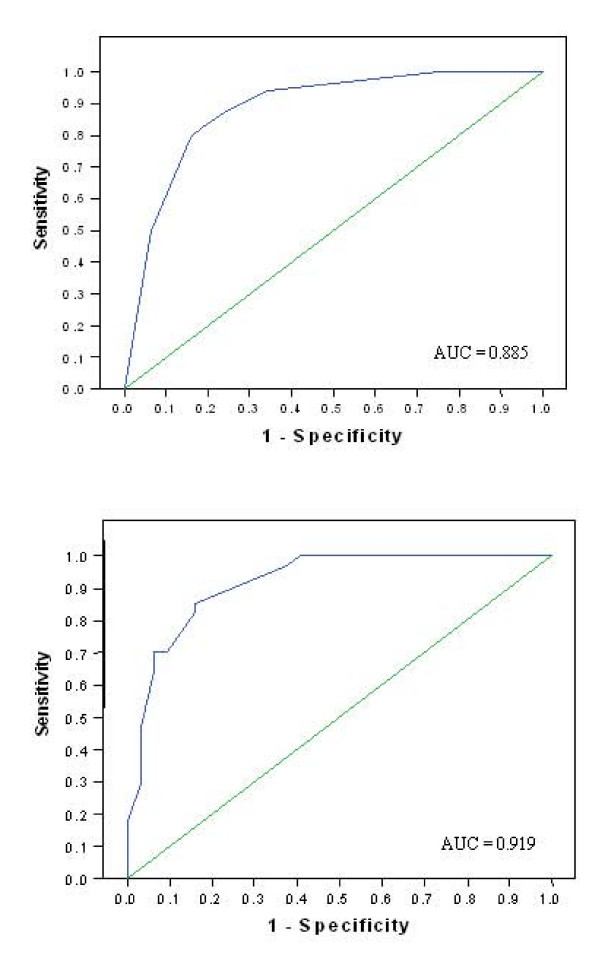
**Receiver operating characteristic curves at day 1 (2a) and day 3 (2b) of prediction models for bilateral absence of early cortical responses to SSEPs**. (2a) The prediction model was based on following predictors at day 1: myoclonus, extensor or absent motor responses to pain and absence of corneal reflex. (2b) The prediction model was based on following predictors at day 3: extensor or absent motor responses to pain, myoclonus, absence of pupillary and corneal reflexes and a malignant EEG.

As shown in Figures 2a and 2b respectively, the ROC curves for unfavorable result of SSEPs indicated a score ≥ 88.5 points with a positive predictive value of 100% and a sensitivity of 18% (95% CI = 8–27) at day three, but failed to determine a score with a positive predictive value of 100% at day one. The scores with the highest specificity and sensitivity were ≥ 28.5 points (day one; Sp = 75% [95% CI = 65–85], Se = 88% [95% CI = 80–96]) and ≥ 40.5 points (day three; Sp = 84% [95% CI = 76–93], Se = 85% [95% CI = 77–94]), with a positive predictive value of 79% and 85%, respectively. At days one and three, these scores predicted absence of early cortical responses to SSEPs with a false positive rate of 21% (day one) and 15% (day three).

### Score predictive of unfavorable outcome (GP-CPC 4 or 5)

**Figure 3:**
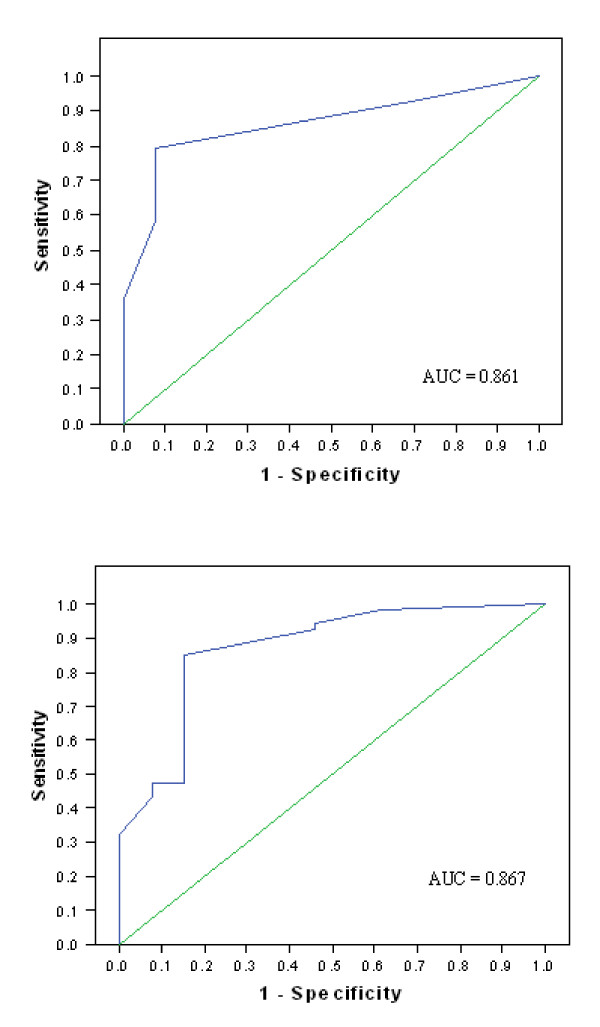
**Receiver operating characteristic curve of prediction models for unfavorable outcome at day 1 (3a) and day 3 (3b)**. (3a) The prediction model was based on following predictors at day 1: myoclonus, extensor or absent motor responses to pain and absence of corneal reflex. (3b) The prediction model was based on following predictors at day 3: extensor or absent motor responses to pain, myoclonus, absence of pupillary and corneal reflexes and a malignant EEG.

As shown Figures 3a and 3b, the ROC curves for unfavorable outcome determined a score ≥ 52 points at day one and a score ≥ 69 points at day three, with a positive predictive value of 100% and a sensitivity of 35.8% (95% CI = 24–47) and 32% (95%CI = 21–43), respectively. An unfavorable outcome was predicted in more than a quarter of the studied population with no false positives. The scores with the highest specificity and sensitivity were ≥ 22 points (day one; Sp = 92% [95% CI = 86–99], Se = 79% [95% CI = 70–89]) and ≥ 32 points (day three; Sp = 85% [95% CI = 76–93], Se = 85% [95% CI = 76–94]) with a positive predictive value of 98% and 96%, respectively. The presence of two or more predictors at day one or day three predicted an unfavorable outcome with a false positive rate of 2% and 4%, respectively.

### Contribution of the score to predict the outcome among subjects with a favorable result of SSEPs

**Figure 4:**
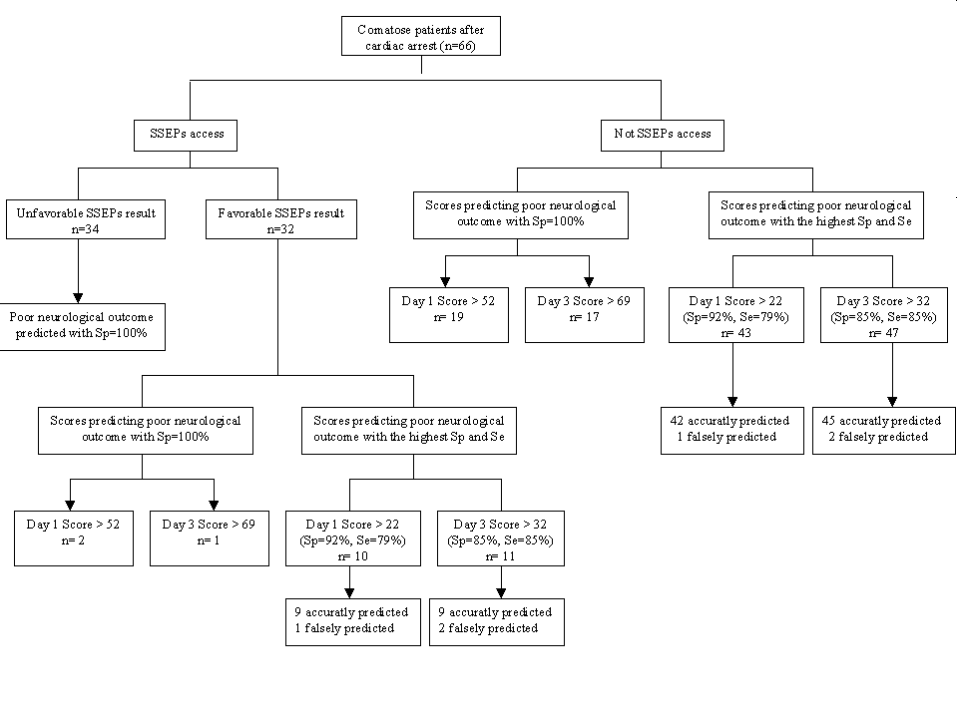
**Contribution of scoring system to predict a poor neurological outcome regarding SSEPs access**. * Sp: Specificity. Se: Sensitivity.

Among 32 patients with a favorable result of SSEPs, 19 patients had an unfavorable neurological outcome at six months (Figure 1). As shown in Figure 4, the scoring system with 100% specificity at day one (score ≥ 52 points) and at day three (score ≥ 69 points) predicted death or vegetative state in two and one patients, respectively. At day three, the score (≥ 32 points) with the highest sensitivity and specificity accurately predicted an unfavorable outcome in 9/19 (47%) patients, but failed in two.

## Discussion

Anoxic coma following cardiac arrest is a common problem with great ethical, social, and legal consequences. To our knowledge, except for unfavorable SSEP results, predictors of unfavorable outcome with a 100% specificity and a high sensitivity are lacking [1,11]. We have identified an early clinical and electrophysiological score that has 100% predictive value of unfavorable result of SSEPs. In addition, we complemented this result with a score predictive of a poor neurological outcome (death or vegetative state) in more than two-thirds of patients, with a low false positive rate (4%) at day three. These results may have important implications in determining the level of care to be provided three days after cardiac arrest, if SSEPs cannot be performed.

SSEPs are considered as the most accurate predictor of an unfavorable outcome in survivors after a cardiac arrest [1,5,8,12-17]. SSEP assessment is superior to clinical or EEG tests in terms of low false positive rates and high prevalence of abnormal test results [8], and the results of SSEP assessment are less affected by metabolic changes and sedative drugs than are clinical features and EEG readings [26,27]. In addition, SSEPs can be used in patients treated with hypothermia [16,28,29].

Our study confirmed the poor likelihood of awakening in patients who remained comatose after cardiac arrest, as 80% of our patients never regained consciousness. This result is consistent with results of previous studies that included only patients who remained unconscious 24 hours after cardiac arrest [2,3,5,8,17,30].

We found that 51% of patients had no early cortical responses to SSEPs, in agreement with previous reports [5,8,17,30]. Furthermore, the presence of early cortical responses to SSEPs was a poor predictor of a favorable outcome as previously reported [4,5,8]; indeed, only 40% (13/32) of our patients with a favorable result of SSEPs made a good recovery.

Because the results of SSEPs demonstrate 100% specificity as a predictor of poor outcome in half of patients in coma patients after a cardiac arrest [1,5,8,12-17], we wanted to identify predictors of unfavorable SSEP result as potential substitutes for SSEPs, in settings without access to SSEPs. At day three, we identified a score (≥ 88.5 points) based on factors that were independently associated with an unfavorable SSEP results (Table 2) with 100% positive predictive value but low sensitivity (18%). In clinical practice, this score could allow early identification of a subgroup of irrecoverable patients for whom continued intensive care could be considered futile. In contrast, the scores with the highest specificity and sensitivity at days one and three could allow early identification of a large fraction of patients with unfavorable SSEPs results. However, the high false positive rate of these scores limits their use as potential substitutes for SSEPs, particularly when considering early withdrawal of intensive treatment.

Regarding poor neurological outcome, the score (Table 2) at day one and day three predicted death or vegetative state with 100% of specificity in one-quarter of our patients, suggesting that a definitive pejorative prognosis could be established early in a subset of patients for whom intensive treatment could be withdrawn. These scores combining simple clinical signs and EEG patterns, had a lower sensitivity than an unfavourable result of SSEPs alone. However, in patients with a favourable result of SSEPs, this scoring system could help in the identification of a subgroup of irrevocable patients.

However, the retrospective, single-center design of this study and the relatively small number of patients studied could limit the interpretation and the clinical relevance of our data. Another aspect that could limit the applicability of these data is that early withdrawal of treatment for patients with poor prognoses could result in a self-fulfilling prophesy of poor outcome. However, all of our patients were actively supported at least until SSEPs were assessed, and only a lack of bilateral cortical responses to SSEPs led to active care withdrawal.

## Conclusion

We developed a scoring system based on a combination of early clinical and EEG factors that predicts with certainty an unfavorable result of SSEPs or a poor neurological outcome with a great accuracy in a subset of comatose survivors after a cardiac arrest. This scoring system had a lower sensitivity than an unfavourable result of SSEPs alone to predict death or vegetative state. However, this scoring system could assist clinicians, in settings without access SSEPs or when an early cortical response (N20) to SSEPs is present, in the early identification of a subgroup of irrecoverable patients for whom intensive treatment could be regarded futile and only palliative care could be given. This strategy needs to be confirmed by larger prospective trials.

## Abbreviations

EEG: Electro-Encephalography; GP-CPC: Glasgow-Pittsburgh Cerebral Performance Category; NSE: Neuron-Specific Enolase; SSEPs: Somatosensory Evoked Potentials; ROC: Receiver Operating Characteristics; Se: Sensitivity; Sp: Specificity; PPV: Predictive Positive Value.

## Competing interests

The authors declare that they have no competing interests.

## Authors' contributions

CD and DG initiated the study, and the design. OE reviewed all EEGs and SSEPs. JJP, CG and CD performed the statistical analysis and were involved in the interpretation of the results. CD wrote the manuscript, and JJP and PC helped to draft the manuscript. OE, DDC, MR, BB and PC contributed to the conception and design of the study and revision of the manuscript. All authors read and approved the final manuscript.

## Pre-publication history

The pre-publication history for this paper can be accessed here:



## Supplementary Material

Additional file 1 **EEG pattern according to the classification system of Synek **[20,21]. The data provided represent the EEG pattern according to the classification system of Synek. Click here for file

Additional file 2 **Neurological outcome according to the Glasgow-Pittsburgh Cerebral Performance Category (GP-CPC) **[22]. The data provided represent the five grades Glasgow-Pittsburgh Cerebral performance category (GP-CPC) scale Click here for file
