# Supplementary material for: A clinical and EEG scoring system that predicts early cortical response (N20) to somatosensory evoked potentials and outcome after cardiac arrest
Source: BMC Cardiovasc Disord. 2008 Dec 4;8:35. doi: 10.1186/1471-2261-8-35 (PMC2630986; doi:10.1186/1471-2261-8-35)
Supplement: Additional file 1 — EEG pattern according to the classification system of Synek [20,21]. The data provided represent the EEG pattern according to the classification system of Synek. [file 1471-2261-8-35-S1.doc]

**Additional file 1**: EEG pattern according to the classification system of Synek [20-21]

*Benign*: near normal; rhythmic theta, reactive; frontal rhythmic delta, reactive or non reactive; and spindle coma.

*Uncertain*: mixed theta and delta, non reactive; dominant delta, reactive or non reactive; alpha-coma, reactive; and epileptiform discharges on base of diffuse delta.

*Malignant*: low amplitude delta (<50 μV), nonreactive; burst suppression; suppression (< 20 μV), alpha/theta coma non reactive; and epileptiform discharge with burst suppression.
