# Supplementary material for: A clinical and EEG scoring system that predicts early cortical response (N20) to somatosensory evoked potentials and outcome after cardiac arrest
Source: BMC Cardiovasc Disord. 2008 Dec 4;8:35. doi: 10.1186/1471-2261-8-35 (PMC2630986; doi:10.1186/1471-2261-8-35)
Supplement: Additional file 2 — Neurological outcome according to the Glasgow-Pittsburgh Cerebral Performance Category (GP-CPC) [22]. The data provided represent the five grades Glasgow-Pittsburgh Cerebral performance category (GP-CPC) scale [file 1471-2261-8-35-S2.doc]

**Additional file 2**: neurological outcome according to the Glasgow-Pittsburgh Cerebral Performance Category (GP-CPC) [22]

*GP-CPC 1*: conscious, alert, and oriented with normal cognitive functions

*GP-CPC 2*: conscious and alert with moderate cerebral disability

*GP-CPC 3*: conscious with severe disability

*GP-CPC 4*: comatose or in persistent vegetative state

*GP-CPC 5*: certified brain death or dead by traditional criteria.
